# A Dialogue-Based System with Photo and Storytelling for Older Adults: Toward Daily Cognitive Training

**DOI:** 10.3389/frobt.2021.644964

**Published:** 2021-06-29

**Authors:** Seiki Tokunaga, Kazuhiro Tamura, Mihoko Otake-Matsuura

**Affiliations:** RIKEN Center for Advanced Intelligence Project (AIP), Saitama, Japan

**Keywords:** dialogue system, storytelling, question-answering, cognitive training, daily use, home-based experiment

## Abstract

As the elderly population grows worldwide, living a healthy and full life as an older adult is becoming a topic of great interest. One key factor and severe challenge to maintaining quality of life in older adults is cognitive decline. Assistive robots for helping older adults have been proposed to solve issues such as social isolation and dependent living. Only a few studies have reported the positive effects of dialogue robots on cognitive function but conversation is being discussed as a promising intervention that includes various cognitive tasks. Existing dialogue robot-related studies have reported on placing dialogue robots in elderly homes and allowing them to interact with residents. However, it is difficult to reproduce these experiments since the participants’ characteristics influence experimental conditions, especially at home. Besides, most dialogue systems are not designed to set experimental conditions without on-site support. This study proposes a novel design method that uses a dialogue-based robot system for cognitive training at home. We define challenges and requirements to meet them to realize cognitive function training through daily communication. Those requirements are designed to satisfy detailed conditions such as duration of dialogue, frequency, and starting time without on-site support. Our system displays photos and gives original stories to provide contexts for dialogue that help the robot maintain a conversation for each story. Then the system schedules dialogue sessions along with the participant’s plan. The robot moderates the user to ask a question and then responds to the question by changing its facial expression. This question-answering procedure continued for a specific duration (4 min). To verify our design method’s effectiveness and implementation, we conducted three user studies by recruiting 35 elderly participants. We performed prototype-, laboratory-, and home-based experiments. Through these experiments, we evaluated current datasets, user experience, and feasibility for home use. We report on and discuss the older adults’ attitudes toward the robot and the number of turns during dialogues. We also classify the types of utterances and identify user needs. Herein, we outline the findings of this study, outlining the system’s essential characteristics to experiment toward daily cognitive training and explain further feature requests.

## 1 Introduction

Many societies around the world are aging rapidly, and thus, maintaining quality of life (QOL) among their older adults, who often suffer from social isolation and loneliness, has become an important concern. Older adults need positive social interactions and communication to stay mentally and emotionally fit (Fingerman et al., 2019). These social activities involve the activation of higher cognitive functions and have the potential to prevent cognitive decline among the elderly (Krueger et al., 2009). Research has determined that communication with others balances the sympathetic nerve activity, leading to enhanced emotional well-being (Seeman et al., 2001). Furthermore, several psychological studies have reported that the task of conversation is cognitively simulating (Dodge et al., 2015). Encouraging older adults to take part in conversation is a promising intervention that includes various cognitive tasks such as attention, speech recognition, verbal comprehension, memory, planning, verbal generation, and speech generation (Ybarra et al., 2008).

Applications of gerontechnology have been investigated since the late 1980s to address the problems of older adults, exploring ways of enabling them to live healthy, independent, socially engaged lives for as long as possible (Graafmans and Taipale, 1998). Shishehgar et al. (2019) categorize the issues faced by older adults and how robots negotiate these issues, which include social isolation, dependent living, physical or cognitive impairments, mobility problems, poor health monitoring, lack of recreation, remembering problems, and fall problems. Miyake et al. (2020) have developed a monitoring-bedside robot that aims to prevent falls, which are otherwise common among the elderly. The robot talks to the bed occupant to prevent them from arising from the bed before a care worker arrives. Broadbent et al. (2014) have developed a robot that focuses on healthcare, with functions such as reminders to take medication and involve cross-over trials on the older adults who live in the retirement village. There are several studies on cognitive training for older adults. Yaghoubzadeh et al. have proposed a dialogue system that is specialized for cognitively impaired users (Yaghoubzadeh and Kopp, 2017). Miura et al. (2019) have proposed and evaluated a chat-based application that encourages older adults to check both their health and mental status by themselves. The coimagination method (CM) is a kind of group conversation support method to maintain cognitive function in which listening and speaking are moderated by rules (Otake et al., 2011; Otake-Matsuura, 2018). A cognitive intervention program named PICMOR, which is based on CM with the guidance of robotic moderators, had positive effects on healthy older adults’ cognitive function (Otake-Matsuura et al., 2021). In CM’s rule, each participant is given a specific theme common in an experiment (e.g., favorite food) and the participants take a photo of the theme. Then, conversations are conducted with strict time-keeping based on the CM’s rule. After the conversation session, each participant summarizes their discussion, with a short sentence of not more than 200 characters in Japanese. The summary is named as a story (Tokunaga and Otake-Matsuura, 2019). Van Patten et al. (2020) have suggested that robots that assist older adults at home and help with cognitive aspects of life have become increasingly crucial because most older adults would like to keep living in their own houses rather than in nursing facilities.

Despite the potential benefits of using dialogue robots, only a few studies have reported on their positive effects on cognitive function. To elucidate the intervention effects of experiments with robots, there is a need to reproduce the experiments with some changes. For instance, it has been reported that a doll-type communication robot has a positive effect on cognitive function in older adults living alone (Tanaka et al., 2012). However, in general, it is difficult to maintain experimental conditions when monitoring communication between dialogue robots and participants because it often depends on the participants’ characteristics. Some older adults may forget what to talk about because of the lack of context surrounding the dialogue. These situations occur not only for dialogue robots but also other robots who interact with older adults in their homes.

To address these challenges, we propose a dialogue-based system that uses photos and storytelling and aims to provide daily cognitive training for healthy older adults. Then, we developed three functional and nonfunctional requirements to cope with the three challenges. The requirements include the feasibility of daily use in the participants’ homes. The steps of the development process involved developing an integrated system based on a cloud computing system. The client system includes a user-centered designed application run on a tablet and a dialogue robot. The cloud system consists of a web-based dialogue–session–schedule–management system, a dialogue, and a logging-management proxy server system, a dialogue system with question-and-answer datasets, stories, and a photo management system. We also describe how to prepare datasets of question-and-answer (QA) pairs. We have proposed some of the subsystems in our previous articles on dialogue and log management proxy server system (Tokunaga and Otake-Matsuura, 2019), a robot (Tokunaga and Otake-Matsuura, 2020), and a set of prototype-based systems consisting of a user-centered designed application, a robot, and a dialogue system (Tokunaga et al., 2019). However, we have not reported other subsystems or evaluated the whole integrated system with older adults. Hence, this is the first study to assess the integrated approach toward daily cognitive training. To confirm whether the proposed system concept is suitable for the target users who are healthy older adults, we conducted three-step user studies; prototype-based, laboratory-based, and home-based. Each experiment was designed with one of the following goals in mind: first, the prototype-based study is designed to evaluate the robot’s acceptance rate and the proposed system. The laboratory-based user study is also designed to confirm the usability of the fully proposed system among older participants and what type of user’s utterance is collected by annotation. The home-based experiment ensures that the proposed approach is ready for home use by older adults, which is the primary purpose of our research.

## 2 System Requirements for In-Home Use of the Dialogue System

Our research objective is to develop methods that contribute to the cognitive health of older adults in their homes. It is necessary to design a system for daily support because older adults stay for longer durations of time in their houses. Thus, we devised the aforementioned objective, and daily use became a key characteristic of the solution we intended to develop. This study explains in detail some of the challenges of developing a home-based dialogue system and describes three essential requirements from functional and non-functional perspectives. Each functional requirement corresponds to an individual challenge. The nonfunctional requirements do not correspond directly to the challenges; however, these nonfunctional requirements support the system’s development overall.

### 2.1 Challenges of Daily Cognitive Training Using Dialogue System

#### 2.1.1 C-1: Problems of Undertaking Discussion With Dialogue System Without a Topic

Using a dialogue system and ensuring that users keep talking with it is challenging for older adults. In general, conducting dialogue just like a chat with the system is difficult for older adults and other younger people because it does not provide context for conversation. Compared to humans, a relationship with a dialogue system is up to the participants. Chat-based dialogue systems are ready to receive general utterances but do not suggest what kind of utterances are acceptable. As a result, users of a dialogue system tend to be at a loss. Since it is challenging to prepare answers to any questions, the dialogue system’s response often does not respond much to the user’s utterance, which might decrease the user’s motivation to keep on talking with the system.

#### 2.1.2 C-2: Difficult Experimental Conditions in Daily Life

Conventional dialogue systems have difficulty applying a traditional dialogue system due to setting experimental conditions (e.g., time duration and schedule). One reason is the lack of a detailed scheduling feature that sets the experiment duration and how long it takes for one dialogue session, given that the intervention effect depends on their conditions. Furthermore, if the daily experiments are conducted at the participant’s home, we have to consider the participant’s schedules because they have daily plans in their life.

#### 2.1.3 C-3: Challenges of Managing Experiment Conditions Remotely

A home-based experiment is more challenging compared to a laboratory-based study. In a laboratory-based experiment, we can support participants on the spot. Although we can help the participants directly when they worry about using the system in a lab, we cannot help in a home-based experiment and it can be difficult to fully understand the participants’ problems from a remote location. Participants can be supported remotely with some tools such as telephones and video conferences. For our experiments in daily cognitive training in everyday life at home, we were aware that these systems could result in excessive communication between the staff and participants, which may affect the outcome. Thus, without a specialized system that is well managed remotely, such an investigation cannot be conducted.

### 2.2 Functional Requirements

#### 2.2.1 FR-1: Providing a Variety of Contexts for Dialogue

This requirement is targeted at ensuring that the older adults can easily conduct dialogue experiments efficiently, addressing challenge C-1. In a long-term experiment, the lack of dialogue context or a monotonous context becomes a problem. If the contexts change for each session, the users can think of new questions and comments, which should motivate the users to continue the dialogue daily. Therefore, the system should provide a different dialogue topic in each session.

#### 2.2.2 FR-2: Setting the Experimental Conditions

Considering that this is an intervention experiment, the system should have a scheduling feature that overcomes the challenge C-2. Hence, the experimental system should have a feature to set the experimental condition’s time duration and include a start and end time. Our dialogue system is based on CM. The system should pair a photo and a story, and arbitrary session-time. Hence, the system meets this requirement, which enables detailed control of the experimental conditions. Our study is also geared toward daily experiments at home, meaning this feature needs to be implemented as a remote operation.

#### 2.2.3 FR-3: Having Self-Explanatory and Automatically Runnable

Our system was designed for remote experiments; hence, the system should be self-explanatory, providing enough information on what participants should do next. This requirement is set to address C-3. Therefore, the system should meet the requirement that the users should easily understand what to do during the interaction. In a laboratory-based experiment, when the participants worry or become confused about using the system or the robot, we can provide direct advice or answers to any questions on the spot. However, in a home-based experiment, providing guidance will be more difficult because we cannot conduct instruction or help immediately. Therefore, the system should be able to clearly inform the users on what they have to do. User progression in the context of the system should be accompanied by an easy-to-understand advice message. We also believe that to conduct daily experiments, especially for older adults, from being confused about the operation of the system, we need to minimize the number of operations from the user side as much as possible. Therefore, we have also designed the system to be capable of automatic execution as a functional requirement.

### 2.3 Nonfunctional Requirements

#### 2.3.1 NFR-1: Usability for Older Adults

The UI of the application should be carefully considered to be appropriate for older adults. For example, the fonts and photos’ sizes should be big enough for older adults to discern. In addition, the CM domain’s necessary information, which includes limitations regarding the time and the photo, should appear clearly on the screen. Hence, the system should display essential information regarding question-answering time and the photo that are easy for the users to recognize. Moreover, the system should be able to respond to user utterances and interactions within a short amount of time (e.g., few seconds), because an exceedingly late response will be difficult to recognize for elderly users. In contrast, an unfriendly UI may be burdensome for older adults.

#### 2.3.2 NFR-2: Well-Managed Error Handling

Because the experiments are conducted remotely, the system should run toward long-term experimentation, the system should be carefully designed to cleanly handle errors such as network errors or other unexpected system errors. Otherwise, during a home experiment, the visible error statuses or unpredictable behaviors will be a burden to users, especially the elderly. If the system encounters such errors, it should be able to handle these cleanly, such as by showing messages that are suitable for the user and by exiting gracefully to avoid an invalid system state.

#### 2.3.3 NFR-3: Remote System Operation and Monitoring

During the at-home experiment, system operation (e.g., dialogue session) and the results (e.g., chat history, operational log) should be delivered remotely to monitor the system, and to that, the participants encounter no trouble during the at-home experiments. Moreover, internet status should be captured from the operator’s perspective when we consider how to develop the apps, which require an internet connection. Logging data should be delivered in real-time because exceedingly long delays are not acceptable from the experiment operator. However, if the operator knows the near-real-time situation of the users, he or she would be able to handle any errors in communication.

## 3 Development of Dialogue-Based System With Photo and Storytelling: DBSPS

### 3.1 System Overview

**FIGURE 1:**
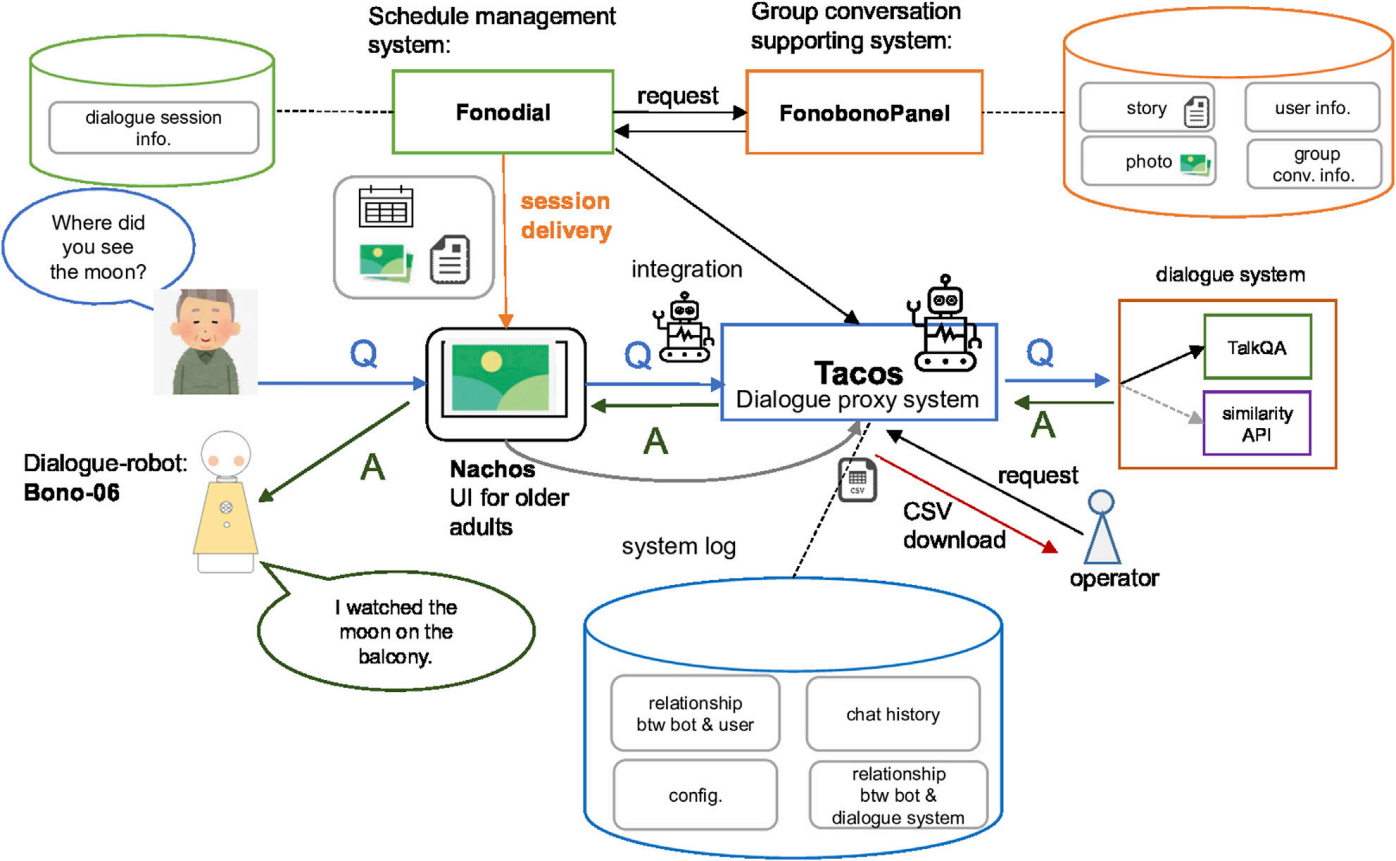
Diagram for dialogue-based system with photo and storytelling (DBSPS).

Figure 1 shows a system architecture diagram representing the data flow and components of the proposed solution. We named the entire system “Dialogue-Based System with Photo and Storytelling” (DBSPS). On the left side of Figure 1, near the elderly users, is the robot (Bono-06) and a tablet application (Nachos), which displays essential information, that is, photos and experiment time. These are cloud-based client systems. Positioned in the upper center of Figure 1 is a schedule management system (Fonodial) and the upper right box represents the existing system that collects photos and stories for the group conversation (FonobonoPanel). The latter system is originally used to conduct group conversations in CM sessions. However, in this study, we used it as a data source, which provides photo images and story information. In the center of Figure 1 is a proxy server program (Tacos), which is responsible for retrieving the QA between the user and the dialogue system. The system is equipped with features for recording and exporting logs as CSV format files, which will be used as an experiment for data management. Lastly, the box in the middle of the right side represents dialogue systems, which respond to questions from the user. Using the proposed system, participants can talk with the robot, which will present enriched content such as original stories and pictures that encourage the participants to ask questions. The following introduces how the dialogue-based system was developed, and its dataset, and how to design each system to meet the requirements.

### 3.2 Web-Based Dialogue Session Schedule Management System: Fonodial

We designed and developed a web-based dialogue session schedule-management system called Fonodial. This system is responsible for managing and delivering the schedules of dialogue sessions to an application that provides experimental information via the cloud. Fonodial can schedule dialogues in two ways: scheduled and immediately. Either way, the schedule type is provided to another application (Section 3.3). When the dialogues are scheduled (referred to as “scheduled type” from here on), preliminary schedules for daily use of the system are assigned to the participants. For example, if the experiment starts on November 25, at 10:00, then that date can be set as the starting date in the system. The system will then send scheduled data via the cloud, consisting of the session schedule type and dialogue context settings comprised of photos and stories from the existing system. This module aims to meet the requirement FR-2, being ready to set the experimental conditions. In the other scheduling type, the experiment is conducted immediately. The experimental moderator will be able to run the experiment immediately on demand, for example, when the participants are ready to perform the experiment casually, sometimes, or when redelivery is necessary for the experimental session. Moreover, if system issues occur during the investigation, the experimental moderator can use this feature to deliver another experimental schedule for a backup procedure.

### 3.3 UI of Dialogue System for Older Adults: Nachos

**FIGURE 2:**
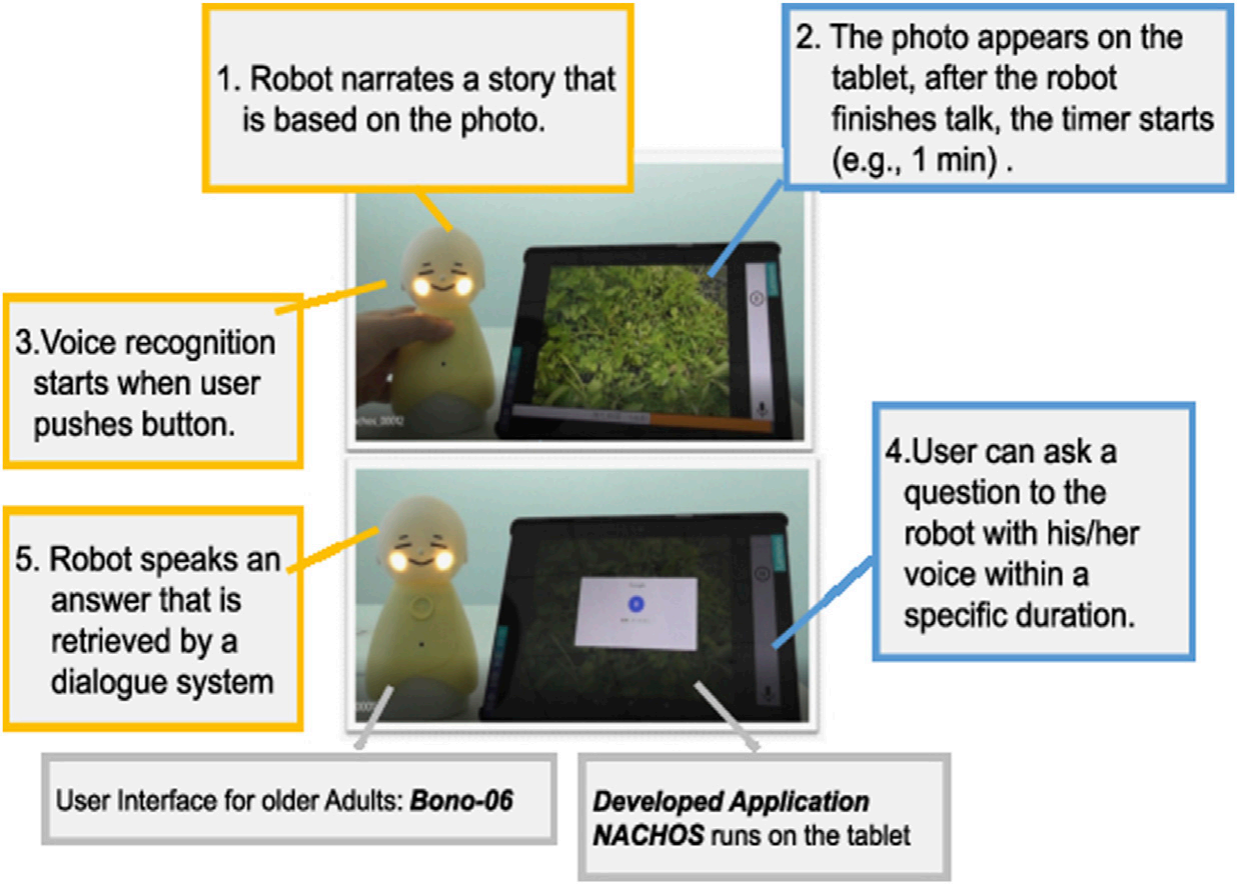
System integration with dialogue robot and nachos.

**FIGURE 3:**
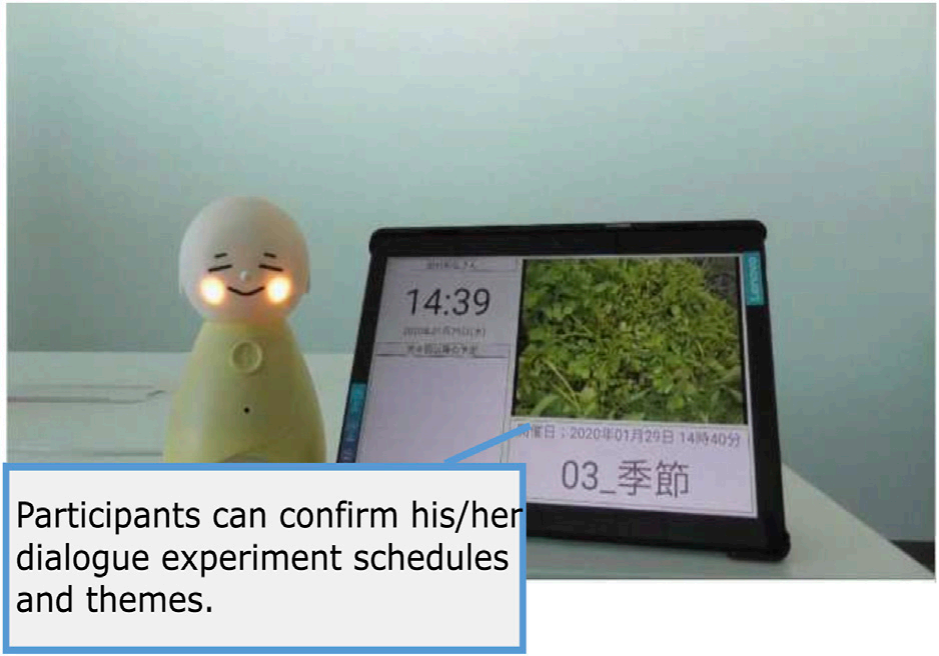
Screenshot of Nachos; the recent schedule appeared for the participant.

Figure 2 represents an original application that we have developed—Native Application of Coimagination-driven Human-centered Orchestration System (Nachos)—based on a basic system concept derived from our previous work (Tokunaga and Otake-Matsuura, 2019). Nachos is an android application that is designed to provide rich dialogue interaction with storytelling and QA experience for older adults. We tried to reduce cumbersome screen-based operations (e.g., a tap) for older adults. The system is integrated with the robot, Bono-06 (Section 3.5). Its main feature provides a QA experience for older adults with a photo and storytelling within a specific duration (e.g., 1 min). This feature corresponds to providing contexts for dialogue (Section 2.2.1). Nachos is designed to receive the dialogue schedule from Fonodial via the internet. Based on the scheduled time, it automatically runs its program; therefore, the experiment can be conducted wherever an internet connection is available, such as in the home of a participant. These scheduled features are designed to meet the requirement of setting experiments remotely (Section 2.2.2). As the nearest scheduled time approaches, Nachos starts the dialogue session with notifications to the users via voice messaging. This module explicitly encourages the participants to do the actions, and following procedures run automatically to meet the requirement of FR-3 in Section 2.2.3. The voice message will include a text of a story and prefaces consisting of the date and the topic of its story. For example, consider one example of prefaces: “Today is October 1st, let’s have a conversation. Today’s topic is a favorite thing.” Then, the robot narrates the story based on the photo (Figure 2), 1) as: “About 20 years ago, I bought a tray at an overseas airport.” When the robot finishes giving a story, the robot encourages the participants to ask some questions, “Could you ask me some questions?”. Then, the time for questions starts. We can set an arbitrary period, for instance, 1 min. During question time, the system accepts questions. The timer starts according to the preset period (Figure 2). 2) Voice recognition starts when a user pushes the button (Figure 2). 3) Users can ask a question to the robot in their voice within a specific duration (Figure 2). 4) The robot speaks an answer that is retrieved by a dialogue system (Figure 2). 5) After that, when the QA time expires, the robot speaks the last message that the session has completed, “Thank you very much for your questions.” This system is also designed with a reporting feature, which provides easy and detailed traceability on what happened for individual users during the experiment. The system communicates with other systems via the Internet to transmit data such as the resulting transcript of user utterances and the system log (e.g., robot operations, Bluetooth disconnection). This feature is designed to meet the requirement of NFR-3 in Section 2.3.3. On a standby screen represented in Figure 3, the participant’s recent schedule is shown on the right side. Using this feature, participants could confirm theirschedules beforehand^1^.

### 3.4 Dialogue and Log Management Proxy Server System: Tacos

We designed and developed the server program, called the Text-Oriented Artificial Chat Operation System (Tacos), which works as the mediator between Nachos and dialogue systems (Tokunaga and Otake-Matsuura, 2019). Tacos can receive both chat and system logs remotely on the Internet; hence, the system operator could confirm the system’s log remotely via a web interface designed to provide remote monitoring for NFR-3 (Section 2.3.3). Its basic procedure is as follows. Tacos receives a question (Q) from the elderly participants through Nachos, then asks the dialogue system, which produces an answer (A) that returns to Nachos (see Figure 1 where Q and A are illustrated with the arrows). Tacos introduces the concept of dialogue bot in its logic, which manages common information for the dialogue system, such as unknown utterances like “I cannot understand you.” The dialogue bot manages a logical space in the dialogue system, which is referred to as workspace. The workspace manages to learn data that are trained for a specific topic (e.g., “favorite thing”) to provide an appropriate response for the given topic, which is necessary to provide an appropriate answer to the questions considering the given contexts based on the stories. Thus, those features satisfy the FR-1 (Section 2.2.1). Nachos and Tacos exchange system information to conduct an appropriate dialogue in the dialogue session. The data flow proceeds as follows. First, after Nachos starts its dialogues session for a specific topic (e.g., favorite thing), it exchanges information from the dialogue bot with Tacos. Afterward, Tacos switches its workspace dynamically to favorite thing. Tacos is now ready to receive the questions for the favorite theme thing. With this switching functionality, the system can deal with multiple contexts rather than a single context, which corresponds to FR-1 (Section 2.2.1). During the experiment, Tacos receives both chat and system logs consisting of the user’s utterance, recording time, error log, etc. Therefore, an experimental operator can observe whether the error has occurred or not in a remote. The operator could download those results in the CSV format. Thus, we can collect the results remotely using this feature. Tacos also supports a multiple-dialogue system feature, which can adapt to the experiment and that provides flexible choice and sometimes fault tolerance (e.g., if one system is down for some systematic reason, then Tacos can switch to another alive dialogue system). This error-handling feature meets the requirement defined in NFR-2 (Section 2.3.2). Currently, Tacos supports TalkQA, a commercial dialogue system, and API, which was originally developed by our team, as shown in the middle right side of Figure 1.

### 3.5 Dialogue Robot: Bono-06

**FIGURE 4:**
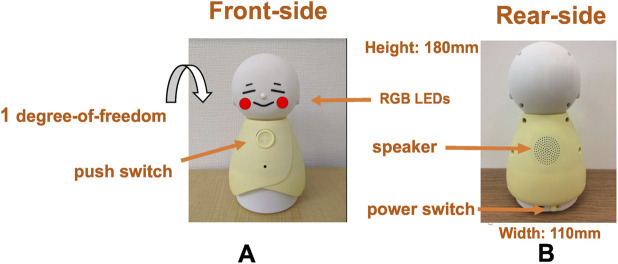
Appearance of dialogue robot Bono-06. **(A)** The front-side and its physical features. **(B)** The rear-side and its size.

**FIGURE 5:**
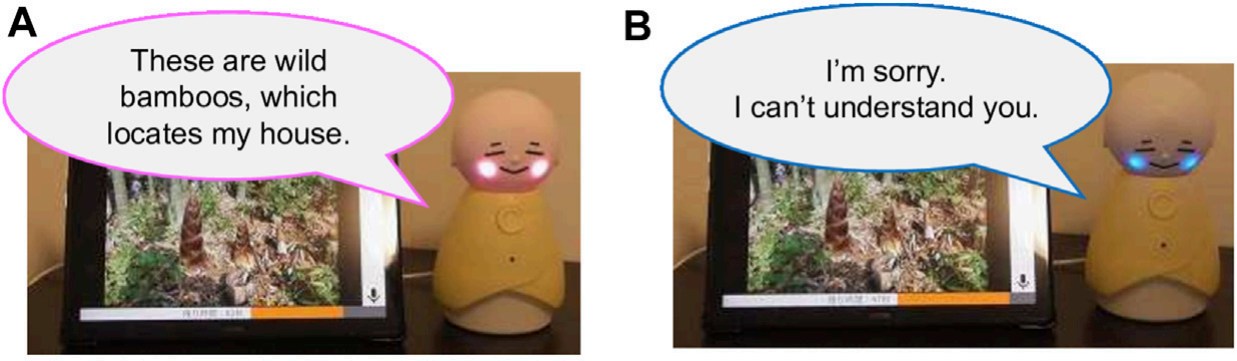
Facial expression patterns of Bono-06. **(A)** Bono-06 replies, confident answer to the user. **(B)** Bono-06 replies, unconfident response to the user.

We developed an original robot called Bono-06 (Tokunaga and Otake-Matsuura, 2020), which aims to provide intuitive interactions and represent the system’s current status toward NFR-1 (Section 2.3.1) for older adults. The specifications of the robot (see the right side of Figure 4) are 180 mm height, 110 mm width, and 181 g total weight. Thus, the robot can be placed on a flat table at home for daily use. The robot can be carried by hand. The robot can be operated via Bluetooth, which could be connected to a smartphone or a tablet. Thus, the participants can put the robot and the tablet at a certain distance, which enables better adaption to the participants’ home conditions. Bono-06 has a physical push switch, which can be used for user intuitive interaction without cumbersome operations. Currently, we used this switch as a trigger for starting speech recognition (see the left side of Figure 4). The robot also has a power switch for an easy on/off switch for the elderly (see the right side of Figure 4B). Bono-06 has RGB LEDs in its cheek (see the left side of Figure 4) to express both the system’s impression and status. The LEDs are currently used for status management via Bluetooth, wherein blinking indicates that connection is not established with its application (Nachos), whereas the yellow LEDs lights indicate a successful connection to Nachos. Moreover, we used the LEDs to also indicate the status of confidence in the dialogue. The pink LEDs lights are confident to respond to the question from the elderly participant (see Figure 5A), whereas the blue lights indicate that the system does not have enough confidence of an answer for the user (see Figure 5B). This intuitive status management with LEDs helps the users and provides easier support during the experiment, which corresponds to FR-3 (Section 2.2.3). Furthermore, we designed Bono-06 as a passive-based system, mainly controlled by an external program (e.g., changes in the color of LEDs, nodding). Thus, it does not require a complex operating system, and because of its simple mechanism, the robot can work robustly for everyday use. In this study, we have integrated Bono-06 and Nachos (Section 3.3) for the experiment.

### 3.6 Stories and QA Pairs

We collected stories and photos from the original system called Fonobonopanel, which is shown in the upper right of Figure 1. The system had features designed to store both photos and stories from participants and provide that information via an external interface with the HTTP protocol to integrate with other systems. Therefore, in this study, we used the system as the data source provider to obtain stories and photos. We then applied these data sets as the dialogue’s context toward FR-1 in Section 2.2.1. We selected 36 stories from two older adults who made the other participants laugh a lot based on previous research (Kikuchi, 2017). The following are the 12 themes that were selected from the previous user study: 1) favorite things, 2) neighborhood landmarks, 3) I try to get off the train at a station that I seldom use, 4) favorite foods, 5) for my health, 6) found on a 10-min walk, 7) saving energy, 8) funny stories and mistakes, 9) things to get rid of, 10) exercising one’s ingenuity in life, 11) feeling the season, and 12) starting something new. Each of the 12 themes had three stories. We collected enough stories to provide three stories to participants per week, which will take three months until all of the stories are used up. We anonymized the original stories to protect the privacy of the storytellers and condensed the abstraction of the sentences to allow other listeners to understand the stories more easily. The latter is required because sometimes an exceedingly detailed location name may cause difficulty for the participants who do not know that location. Specifically, we revised the story’s length to be spoken with text-to-speech technology within a duration of 30–40 s. The duration was adjusted to be as long as one of the tasks in WMS-R (Wechsler, 1987), which aims to assess for the delayed memory cognitive function by testing whether the participant could remember sentences spoken by a human operator. We developed a persona for the storyteller, which is then installed on the robot (Section 3.5). This persona is based on the two men who provided the original stories. Thus, the persona was characterized as a 70-year-old male with hobbies such as fishing. Finally, we generated pairs of QAs to develop a QA-type exchange dialogue system. We referred to the 36 stories that we collected to generate the dataset. We then generated a fixed number of pairs of QAs using crowdsourcing based on rules such as, “When you create QA pairs, please first select a question that refers to the provided story and then create an answer that corresponds to the question you have created.” The procedures of the development of persona and dataset are described in detail in our previous article (Tokunaga et al., 2019). Currently, we have collected about 550 QA pairs for each story.

## 4 Experiment

We conducted three types of experiments to confirm whether the proposed method works as well as we thought with targeted subjects, via a step-by-step approach. First, we evaluated a prototype-based system to confirm our proposed system’s proof of concept (Section 4.1). Second, we assessed the fully developed systems to verify their usability and feasibility. We also evaluated responses from the dialogue system with annotations by a human annotator (Section 4.2). Third, we evaluated the system’s feasibility and usability via home-based experiments and conducted interviews to gather feedback from the participants (Section 4.3).

### 4.1 Experiment 1: Prototype-Based Experiment

#### 4.1.1 Method

**FIGURE 6:**
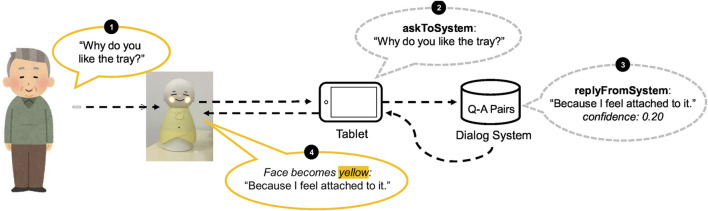
Diagram of prototype system.

**FIGURE 7:**
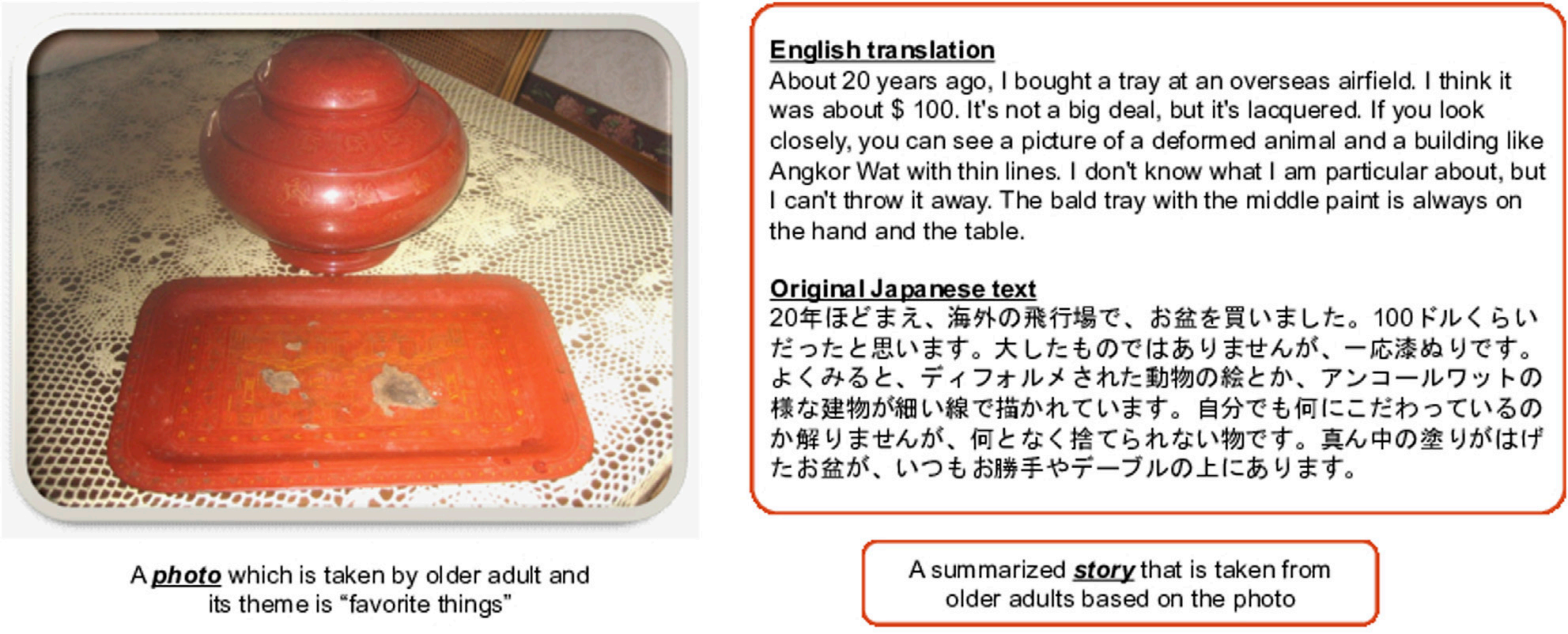
Example pair of photo and story whose theme is favorite things.

We conducted a user study that revealed older adults’ attitudes toward the robot, their ability to remember the stories they experienced, and some technical difficulties with the QA system and the robot. The prototype-based system consists of Bono-06, Android application, and dialogue system (see Figure 6). There were 21 participants, all of whom were healthy older adults. The gender distribution was 12 men and 9 women, and their average age was 75. The settings and results are explained in detail in our previous article (Tokunaga et al., 2019). During the user study, the human operator sat next to the participant to assist if they needed help. Moreover, to keep a detailed record of the user study, we captured a video using a handheld camera. One participant and one human operator conducted this user study. The user study procedure was as follows: the robot (Bono-06) vocalizes a specific story (see the right text of Figure 7), which was generated according to the procedures in Section 3.6. The participant asks a question about the story within 3 min, which is illustrated in Figure 6. The robot speaks the sentence, then the robot receives the participants’ questions before the QA time is over. After the participants spoke with the dialogue-based robot following the aforementioned procedure, we asked them to answer an original questionnaire.

#### 4.1.2 Result

Table 1 outlines the questions that we asked the participants to answer. Most items of the questionnaire had five scales, whereas other items aimed to determine how well each participant could remember the story. Each item of the questionnaire had the following scales between 1 and 5, with a score of such as 3 representing the mid-point between the two extremes: 1) 1: long, 5: very short; 2) 1: definitely understand, 5: do not understand at all; 3) 1: very difficult, 5: very easy; 4) 1: very natural, 5: not natural; 5) 1: very long, 5: very short; 6) 1: very interesting, 5: very boring; 7–9) 1: very good, 5: very bad. There are three main purposes for the questionnaire. In the questions numbered from 1 to 6, we asked about impressions, thus focusing on how participants feel about the storytelling and QA pairs. In the questions numbered from 7 to 9, we asked about the general appearance of the robot, such as size and facial expression. Questions 10 to 12 were relevant to cognitive function tests, such as asking about how much the participant remembers of the original story from the robot. Lastly, we gathered free-form feedback to improve the usability of the robot. The score on the questionnaire number 2 about whether they could understand the story and if it was reasonable. However, the rates of the correct answer being given for questions from 10 to 12 were approximately 50% or less (10: 52%, 11: 43%, and 12: 43%). This result indicates that the participants had difficulty remembering the story from the robot. We cannot judge whether the difficulty stems from the possibility that the speech of the robot may have been difficult for participants to listen to or whether the participants have difficulty remembering the details of the story. The results regarding the appearance of the robot seemed to indicate that its appearance was adequate, according to the participants. In particular, the result for question (7) indicated that the volume of the voice of the robot seemed to be acceptable to the participants because the score was 2.38, which was near one than the middle point of 2.50. Lastly, we demonstrated that providing the story and question-answering during the specific duration format was feasible for the participants.

**TABLE 1:** Questionnaires regarding robot, its story, and responses to questions

Number	Item of questionnaire	Score
1	How do you feel about the length of the story?	2.71
2	Can you understand the content of the story?	2.38
3	How did you feel when thinking of a question?	3.0
4	How did you feel about the response from the robot?	3.19
5	What did you think about the question duration (3 min)?	3.23
6	Did you think the robot’s story is interesting?	2.61
7	What did you think of the volume of the robot’s voice?	2.38
8	What did you think of the size of robot?	2.28
9	What did you think of the facial expression of the robot?	2.33
10	Where did the robot buy the plate?	0.52
11	How much was the plate?	0.43
12	Where did the robot put the plate?	0.43

### 4.2 Experiment 2: Laboratory-Based Experiment

#### 4.2.1 Method

To confirm both the usability and feasibility of the proposed full system (see Figure 1), we conducted a laboratory-based study and compared the results to those of prototype-based (Section 4.1); the difference for this study is that the system has developed completely to integrate with other systems for daily use. Thus, the participants performed the question-answering part of the experiment while looking at a tablet’s photo. Twelve healthy older adults (six men and six women), whose average age was 70, recruited from a silver human resource center, participated in the experiment. Each user study involved three people: one participant, one human operator responsible for supporting the participant in case the participant needed help, and one human experimental collaborator responsible for operating the system to ensure the experiment was conducted smoothly. The participants joined three dialogue sessions during the experiment. The first session was a training session during which participants became accustomed to our system. Before this session was started, participants were introduced to the profile of the robot (Section 3.6). The remaining two sessions were experiments. Each session involved one story and one photo; the question-answer duration was 4 min without counting the storytelling time from the system. Specifically, two themes were used: “favorite things” (Figure 7), and the other was “favorite places in the neighborhood” that are selected from CM’s themes (see Section 3.6). After the experiments, the four participants completed a questionnaire to assess the usability and feasibility. Due to the COVID-19 pandemic, we could not collect the other eight participants’ questionnaire data since the experiments on human subjects were prohibited after the first four participants completed the questionnaires. An annotator annotated the subject’s statements that are aimed to confirm the QA performance with current datasets and dialogue system through a subjective evaluation.

#### 4.2.2 Result

**FIGURE 8:**
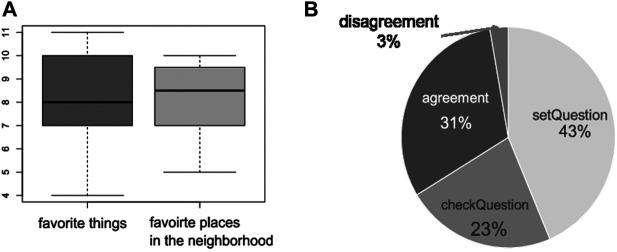
Statistics results in a laboratory-based experiment. **(A)** Statistics of utterances for two topics. **(B)** Proportions of utterances annotated with ISO.

We devised the questionnaires, focusing on the usability of the system based on the system usability scale (SUS), which is regarded as the most practical usability scale (Brooke, 2013). We used a five-point Likert scale questionnaire, where one indicated that the participant “strongly agreed” and five represented “strongly disagreed.” Table 2 outlines the result of the questionnaire showing results for question number seven on the preliminary things learned about the system, and also question number five, which refers to inconsistency, were much better than average. Second, we explained the statistics based on the number of utterances from the participants in each session. We denoted the participants as “users” to keep the article as simple as possible. The total number of user utterances was 198. Figure 8A presents a box plot graph with the number of user utterances during the experiment. In Figure 8A, the left graph represents the first experimental result for the theme is “favorite things,” whereas the right graph represents the result for “favorite places in the neighborhood.” The center of the bold line represents median values; for the first theme, the value is 8, whereas, for the second, the value is 8.5. The average number of questions for the first and second themes was 8.25. The variance for the first theme was 3.35, which is larger than that for the second, which was 2.19. Hence, these results indicate there are no participants who cannot conduct the questions for the system. We evaluated the annotations for both naturalness and compliance with the ISO standard (International Organization for Standardization, 2012). The annotation is produced by one human annotator. The resulting average score for unnaturalness was 0.61, which showed that more than half of the responses were regarded as unnatural. Here, we showed two examples of QA pairs that were annotated unnaturally during this study. Example 1 represents the unnatural QA-pair categorized with unnatural, and example two shows the example data classified with an agreement in the annotation.

**TABLE 2:** Questionnaires about usability of system in a laboratory-based study

Number	Item of questionnaires	Score
1	I can confirm about a question-answering time with this system.	2.75
2	I think that I would like to use this system frequently.	2.75
3	I thought the system was too difficult to use.	2.5
4	I think that I would need the support of a technical person to be able to use this system.	3.25
5	I thought there was too much inconsistency in this system.	3.75
6	I would imagine that most people would learn to use this system very quickly.	2.25
7	I think that I could use this system without getting nervous	3.75
8	I needed to learn a lot of things before I could get going with this system.	1.75
9	I think I can continue to use this system for 1 month without become bored of it.	2.5

Example 1 represents the unnatural QA-pair categorized with unnatural, and the other example 2 shows the example data classified with an agreement in the annotation.

• (Example 1) User: “Where is the shrine? I also would like to know its prefecture.” System: “It’s near a big tree. It protects you from the sun even when it’s hot, the smell of wood calms me down,” confidence: 0.84
• (Example 2) 〈 User: “What is the behind of the plate?”, System: “I feel it’s convenient to carry something and I often tend to put things on it. I feel the plate looks nice.”, confidence: 0.50 〉 User: “I think the plate seems nice too.”, System: “I feel it’s convenient to carry something and I often tend to put things on it (The system responds with the same answer as the previous one.),” confidence: 0.72

For Example 1, the system responded with high confidence (i.e., 0.84); however, the answer seemed a bit strange for the question because the system did not refer to the prefecture. As shown in Example 2, users tend to show agreement to the system response. First, the user asks a question, and then the system answers. Then, the user agrees with the answer. The user’s agreement is processed as the next “question” to the system, but such a question is not prepared as a QA dataset. In this case, the system responds with the same answer to the user. This is because words such as the user’s “plate” matched the data contained in the QA pairs that the system had. As a result, the system’s “answer” to such agreement becomes unnatural, and its confidence is “0.72,”, a score that is relatively high, but in the result, it does not correspond to the user’s utterance.

Figure 8B shows the proportions for different types of user utterances. We annotated with mainly two-dimensional questions and information, with the two main dimensions of subcategories with the ISO standard (International Organization for Standardization, 2012). The former question categories have four subcategories, i.e., propositionalQuestion, setQuestion, checkQuestion, and choiceQuestion. The latter consist of six subcategories: agreement, disagreement, correction, answer, confirm, and disconfirm. As a result, Figure 8B shows utterances from the participants: 43% are categorized as setQuestion, and 23% are categorized as checkQuestion. About one-third of the utterances were annotated as agreement, whereas 2% of utterances were annotated as disagreement. We confirmed that almost all users’ utterances can be annotated based on the metrics mentioned above (91%).

### 4.3 Experiment 3: Home-Based Experiment

#### 4.3.1 Method

We conducted a fully remote experiment to assess whether our proposed system works as expected. Two healthy older adults (two women), whose average age was 74, recruited from a research institute, participated in this experiment. The experimental setup included one participant and a dialogue robot, and a tablet. The experiment was designed for a total of eight days. On the first day, we conducted an explanation session on the system usage and experimental procedure using a video conference tool. All the experimental devices (Bono-06, and the tablet in which Nachos installed) were delivered to the home of each participant before this experiment was started. All the participants received a phone number and a mail address through which they may contact our team in case of a system error or if they require assistance regarding the experiment. On the last day, we interviewed the participants to obtain their impression of the system. For the remaining six days of the program, each participant performed the dialogue user study at their home with our proposed system independently. Thus, before starting the user study, we requested the participants’ schedule and remotely set the dialogue schedule type based on this information (Section 3.2). During each of the six days, the participants conducted two dialogue sessions for a total of 12 sessions; each session had a 4 min question-answering time. The participants then answered the questionnaire used for Section 4.2.2.

#### 4.3.2 Result

The two participants completed all of the twelve dialogue sessions in this study. Three errors occurred during the user study; two due to the robot–tablet connection via Bluetooth, and one was a system issue. We were able to follow the system errors remotely using the reporting and monitoring mechanism (Section 3.3). We addressed errors encountered during the experiments via remote operations (e.g., phone and mail).

Table 3 shows the result of the questionnaire. Most of the items seemed positive, except the number of 4 seemed fair. We confirmed the bad scores through the interview that the main reason was they needed assistance via our proposed system in the experiment that we have reported in the previous statement. Moreover, with the interview after the experiment, we marked the following comments from the participants. The comments are categorized as follows; the comment numbers from 1 to 4 represent feature requests to improve the system, whereas 5 to 8 represent participants’ impressions about the system.

1. If the robot could greet by using a hand, then it would be useful for older adults who have weak legs and are often lying on a bed.
2. It seems better if a bell-like sound is played before a dialogue session is started.
3. I felt the robot lacked the ability to read intent; I thought the answer was just from a machine, not a human.
4. I was glad that I could do sessions anytime, as I decided, and I would like to choose a story from a list-like menu.
5. Combination of a photo and a story seemed nice. I especially thought the photo seemed very elegant.
6. This system might be a good monitoring tool.
7. I had difficulties in asking questions, so I felt the experiment duration seemed long. I felt that my question might be not good enough, as the robot repeated the same answers although I asked different things.

**TABLE 3:** Questionnaires about usability of system in a home-based study

Number	Item of questionnaire	Score
1	I can confirm about a question-answering time with this system.	1
2	I think that I would like to use this system frequently.	2
3	I thought the system was too difficult to use.	4.5
4	I think that I would need the support of a technical person to be able to use this system.	3
5	I thought there was too much inconsistency in this system.	4.5
6	I would imagine that most people would learn to use this system very quickly.	2
7	I found the system very cumbersome to use	4
8	I think that I could use this system without getting nervous	2
9	I needed to learn a lot of things before I could get going with this system.	4
10	I think I can continue to use this system for 1 month without becoming bored of it.	2

## 5 Discussion

### 5.1 Discussion on C-1 and FR-1

#### 5.1.1 Application

In this research, we have proposed the specific dialogue’s context (i.e., a story) within a session. Our research goal was to develop a system for cognitive training that achieves usability and robustness. We have also designed this system to enable older adults—including those unfamiliar with ICT technologies—to use the system as much as possible. If the system did not provide the dialogue’s context, it would have been challenging to talk with the unfamiliar system. Whether people feel difficulty asking robots questions without a dialogue context may depend on personality rather than age. In terms of cognitive training, a training system is necessary for all personality types. We observed that at least 35 of the older adults who participated in this study could ask a certain number of questions —8 to 9 questions on the average— via our system within the specific given duration of —4 minutes. Therefore, our results indicate that older adults who are living independently in their homes could have completed our cognitive training tasks during our experiments regardless of their personalities.

#### 5.1.2 Limitation

As a next step, we need to conduct an experiment with a larger number of participants to confirm the effectiveness of our proposed method for older adults. At the same time, we have not confirmed our results with reference to participants’ personalities; we hypothesize that whether participants conduct experimental tasks passionately is related to the results (i.e., the number of questions asked by participants). Therefore, future work should involve investigation of the personality factor. It may be possible to verify the effectiveness of providing a dialogue’s context by comparing systems with and without such context and investigating each system’s number of questions.

### 5.2 Discussion on C-2 and FR-2

#### 5.2.1 Application

We set experimental schedules, including starting-time, ending-time, and durations, for participants; then, we remotely set the experimental sessions through our system via the participant’s tablets. We also confirmed that the healthy older adults were able to conduct our experiment, which consisted of a total number of 12 sessions. We designed the investigation so that in one day, participants would join two sessions. Therefore, we have confirmed that we can remotely control the experimental conditions, including the starting-time, ending-time, and duration.

#### 5.2.2 Limitation

In this experiment, we evaluated conditions such as frequency for home-based experiments with only a few participants. We completed the entire remote experiment without onsite support for these conditions. Therefore, we need to experiment further to confirm the feasibility and usability of the system with more participants in the near future. In this study, we have confirmed that the experimental conditions are appropriate for healthy older adults at home; as the next step, we also need to conduct an experiment to verify the cognitive training effects on older adults using our system. To conduct the cognitive training experiment remotely, we will need to design the frequency and the experimental period appropriate to the participants; these conditions may affect the results. Hence, when designing such an experiment, we need to consider the participants’ ability to complete the experiment including their original lifestyles and schedules in order to have the effects on their cognitive functions.

### 5.3 Discussion on C-3 and FR-3

#### 5.3.1 Application

We designed our system to run automatically based on the schedules of the participants at home, and we confirmed that it works as designed according to the experimental settings without on-site support. We did not observe that the participants could not understand what to do during the remote experiment; we think that the pre-phase speaking and automatic runnable features derived from FR-3 worked well to prevent this issue. Our system reduces interactions with older adults as much as possible to avoid cumbersome interactions for older adults based on the NFR-1. Therefore a combination of FR-3 and NFR-1 was successful in this home-based experiment.

#### 5.3.2 Limitation

We observed that there were no severe troubles for participants, as we mentioned before; on the other hand, we did receive some requests from the participants during the interviews for a more flexible scheduling feature to enable them to adjust the sessions to their lives. If we conduct a long-term experiment in daily life, participants may face situations in which they are unable to join an experimental session because of harmful health conditions, other sudden phone calls, unexpected visitors, and so on. Therefore, if the system provides some preliminary notifications for the participants (e.g., a reminder feature) and easily adapts to the participants’ situations, that will allow more continuous participation during the daily-life experiment.

### 5.4 Challenges and Requirements Tested Through Experiments

In the prototype-based preliminary experiment, we confirmed that older adults could use the proof-of-concept system, which provides an experimental format comprising a story in the context of the dialogue and a question-answer session of a specific duration. The experimental results suggest that our system solves the problem of C-1 by meeting the functional requirement FR-1. In the laboratory-based experiment, we confirmed that participants could use our system, which also has fully integrated features (scheduling, self-explanatory, and so on) and which was tested using a prototype system in the prototype-based preliminary experiment. We also confirmed that the participants were able to talk with the integrated system. The experimental results suggest that our system solves the problem of C-2 by meeting the functional requirement FR-2. Then, we examined the type of dialogue data collected by the ISO annotation to improve the quality of the question answering. We also asked the participants to assess the consistency of our system with a five-scale questionnaire. As a result of the laboratory-based experiment, the participants were able to use our system, and the collected utterance categorized into four types with the result of annotations. In the home-based experiment, we confirmed that the older adults were able to use the system at home. The experimental results suggest that our system solves the problem of C-3 by meeting the functional requirement FR-3. We completed the home-based experiment without on-site support. We caught a small number of errors during the experiment, but we were able to cope with them remotely, thanks to the application and management system (Section 3.4). Robust operations were achieved via the support of non-functional requirements NFR-1 to NFR-3. After the home-based experiment, we received some requests after the interview, such as a request to make the experimental starting time more flexible in consideration of daily life.

### 5.5 Acceptance and Effectiveness on Older Adults With Various Cognitive Functions

We observed that healthy older adults are the primary targets of our proposed system; hence, our approach and method have not yet confirmed its acceptance and effectiveness for older adults who have declining cognitive abilities. Therefore, we want to clarify, using our approach, the types of participants who might benefit from cognitive training in the near future. In general, it becomes challenging for older adults with low cognitive functions to ask questions based on stories and following instructions because they have difficulty with listening comprehension. Remembering and adjusting their schedules as planned is difficult for them as well. In the future, with further experiments, we hope to define the cognitive level required by participants to use our proposed system. After that, there is the possibility that we may confirm the effectiveness of our cognitive training system for such participants.

### 5.6 Related and Future Works

Similar to our study, Tanaka et al. (2012) have also aimed to achieve cognitive training with dialogue robots. The significant difference between their study and ours is the characteristics of the dialogue system. They used a doll-type robot designed to chat, offer general greetings, sing songs, and so forth, but which was not intended to talk about different dialogue topics. Their system was also not designed to support time management for an experiment. Traum et al. (2015) have developed a storytelling system whose concept is virtual Holocaust survivors. The system aims to offer the knowledge of history through question-answer sessions with the virtual survivor for younger generations rather than real survivors since real survivors are getting old, and it has become difficult to pass down by the conversations. Their approach is similar to ours, because they provide specific storytelling of virtual Holocaust survivors followed by question-answer sessions. In terms of the stories, our system’s originality lies in its provision of different stories as the context, along with the question-and-answer datasets. The Holocaust survivors’ storytelling system was designed for use in a museum, and thereby was expected to provide a one-time-only experience for each visitor. In contrast, our system is designed to be used at home, providing a long-term daily experience for each older adult.

In future work, we plan to improve two points: one is to enhance the performance of the dialogue system, and another is to increase the flexibility of the scheduling. Firstly, we will improve the performance of the dialogue system. We may enhance the naturalness of the dialogue robots’ responses. As a result of ISO annotation, we have confirmed that about one-third of utterances from the participants are categorized as a “not-question” form (e.g., agreement) that may decrease the naturalness of the result. Because we initially prepared the dataset as a question form, this result confirms that participants’ utterances are different from what we expected. There is the possibility of improving the naturalness of the result by preparing a variety of datasets that correspond to users’ utterances from the existing data collected in this study. Secondly, we will increase the flexibility of the scheduling by implementing reminder system. This study confirmed that participants’ situations were sometimes inconvenient when we set the preliminary experimental schedules in the home-based experiment, although we conducted careful system design. To cope with these situations, we want to implement the reminder system that alerts participants to start their dialogue sessions at the start time. After these improvements, we also would like to evaluate our system using a larger number of older adults. In this study, we have primarily evaluated a small number of subjects within a home-based setting; however, we think evaluation with a larger number of experimental subjects could reveal the usability. Then, we will explore possibility of our system to contribute to well-being in daily life. We observed that providing a photo and storytelling experience seemed to create a pleasant experience for the participants during the home-based study. Therefore, we would like to clarify what kind of perspective of our system could contribute to those factors. We would also like to reveal the relationships between the factors mentioned above and participants’ profile data, such as age, gender, and so on.

## 6 Conclusion

This study proposed a system architecture to develop a system for daily cognitive training for older adults, which starts automatically based on scheduling settings and provides a specific dialogue context based on a story and a photo. We have revealed that there are three challenges for conducting daily cognitive training that apply to existing dialogue systems:

• C-1: It is difficult for users to chat with a dialogue robot without knowing the context of the dialogue (e.g., topic).
• C-2: It is challenging to set experimental conditions, such as time durations and schedules, to be used at home.
• C-3: It is not easy to guarantee that users will use the system as planned, even when the experimental conditions are set.

We have found a system that has three key characteristics that are feasible for addressing these challenges:

• We designed a dialogue system that provides a dialogue’s context, such as conducting a question-answer session with a story and a photo.
• We set detailed experimental conditions, such as the topic of the dialogue, start time, and the duration of the experiment, in our system.
• Our system both is self-explanatory and runs automatically to allow the experiment to run smoothly at home.

First, to cope with C-1, we designed a system that provides both a story and a photo (Section 3.6) to provide a dialogue context, the basic concept of which is derived from CM (Otake et al., 2011). Secondly, to address C-2, we set detailed experimental conditions, such as the dialogue’s topic, the start time, and the duration of the experiment, derived from integration with the scheduling system and UI (Sections 3.2, 3.3). Finally, to overcome C-3, we designed our system to have self-explanatory (Sections 3.3, 3.5) and run automatically for a smooth experiment. We have demonstrated how to implement these three characteristics and general dialogue system’s interface as an integrated system, and we have confirmed through experiments that our system can be used by older adults as intended. This is the first study to evaluate an integrated approach to daily cognitive training with older adults. The summary of our experiments is as follows. First, in the prototype-based preliminary experiment, we confirmed that older adults can use the proof-of-concept system, which provides an experimental format comprising a story as the dialogue’s context and a question-answer session of a specific duration. Second, in the laboratory-based experiment, we confirmed that participants were able to use our system, which has fully integrated features (scheduling, self-explanatory, and so on). We also confirmed that about one-third of participants’ utterances are categorized as not-a-question in form. Finally, in the home-based experiment, we confirmed that the older adults are able to use our system in their homes without on-site support.

Our future work will be to conduct experiments to verify the effectiveness of our cognitive training system in a randomized controlled trial using a proposed system with many participants. To accomplish such an experiment, we need to verify the effectiveness of our system with many older participants, proving that the system has enough feasibility for us to conduct an investigation supported by well-managed error handling and monitoring features. With regard to such an experiment, we have identified through this study several improvements required to conduct an investigation smoothly and effectively. The first one is to improve the naturalness of the responses from dialogue robots. The second is to implement a reminder feature, which reminds sessions’ starting time rather than to start on time, so as to be more adjustable to the participant’s lifestyle. The third is to confirm the possibility of contributing to well-being in daily life using the proposed system.

## Data Availability

The original contributions presented in this study are included in the article/supplementary material, further inquiries can be directed to the corresponding author.

## Footnotes

1 We have recorded a demo video that explains how to use the system (see https://www.youtube.com/watch?v=6sCz_-hKngY&t=3s).
